# Association of IL-18 polymorphisms with rheumatoid arthritis and systemic lupus erythematosus in Asian populations: a meta-analysis

**DOI:** 10.1186/1471-2350-13-107

**Published:** 2012-11-15

**Authors:** Shuilian Chen, Feng Jiang, Jiangping Ren, Jiajing Liu, Wei Meng

**Affiliations:** 1 Department of Epidemiology, School of Public Health, Fudan University; Key Laboratory of Public Health Security, Ministry of Education, Shanghai, 200032, China

## Abstract

**Background:**

Interleukin (IL)-18, an important proinflammatory cytokine, plays a potential pathological role in rheumatoid arthritis (RA) and systemic lupus erythematosus (SLE). Studies on the relationship of IL-18 gene promoter rs1946518 (−607A/C) polymorphism, rs187238 (−137G/C) polymorphism with RA and SLE are inconclusive. The aim of this study was to get a more precise estimation of the relationship in Asian populations.

**Methods:**

Meta-analysis was conducted on the associations between the IL-18 (−607A/C and -137G/C) polymorphisms and RA and SLE, using; (1) allele contrast, (2) dominant, and (3) recessive models. A total of 11 studies were included in this study.

**Results:**

For the relationship of IL-18 rs1946518 polymorphism with RA (additive model: OR=0.752, 95%CI=0.562-1.006; dominant model: OR=0.730, 95%CI =0.479-1.113; recessive model: OR=0.537, 95%CI=0.271-1.064) and SLE (additive model: OR=0.684, 95%CI=0.455-1.028; dominant model: OR=0.645, 95%CI=0.368-1.130; recessive model: OR=0.672, 95%CI =0.447-1.010), no significant association with RA and SLE risk can be found under all genetic models in Asian populations. However, significant associations were observed in Chinese population for both RA ((OR=0.688, 95%CI =0.532-0.889) and SLE (OR=0.606, 95%CI =0.396-0.930) under additive model. For the relationship between IL-18 rs187238 polymorphism and RA or SLE, there was no significant association detected in all genetic models, even in Chinese population.

**Conclusions:**

This meta-analysis indicates that the IL-18-607A/C polymorphism may confer susceptibility to RA and SLE in Chinese population, but not all Asians.

## Background

Rheumatoid arthritis (RA) and systemic lupus erythematosus (SLE) are prototypic autoimmune diseases resulting in inflammation and tissue damage. However the etiologies of RA and SLE are not completely clear, a strong genetic component has been supported by genetic and family studies [1,2]. Like most autoimmune diseases, human leukocyte antigen (HLA) genes have been shown to be strongly associated with RA and SLE, and other somewhat weaker but well established associations also have been found with non-HLA genes [3].

IL-18, formerly called interferon (IFN)-γ-inducing factor, is an important proinflammatory cytokine belong to the IL-1 family that is produced by a wide range of immune cells, such as monocytes, activated macrophages and Kupffer cells [4].The imbalance of Th1/Th2 type cytokines plays important roles in the induction and development of several autoimmune diseases, and IL-18 is an important mediator involved in the pathogenesis of Th1- and Th2-mediated diseases including RA and SLE [5,6]. IL-18 can promote Th1-mediated immune response in synergy with IL-12 by producing IFN-γ, and also can induce a type 2 response in the relative absence of IL-12 by inducing the production of Th2 cytokines such as IL-4 and IL-13 [7]. In addition, the levels of IL-18 are significantly elevated in RA and SLE patients compared to healthy controls [8,9]. All the current information suggest that IL-18 may have a potential pathological role in autoimmunity, such as RA and SLE.

The IL18 gene is located on chromosome 11q22.2–22.3 with six exons and five introns [10], and many studies show IL-18 gene promoter polymorphisms are associated with autoimmune diseases (RA and SLE),especially the IL-18 gene rs1946518 and rs187238 polymorphisms. Some studies indicated an association between IL-18 gene polymorphisms (rs1946518 or rs187238) and RA or SLE [11,12], but some studies failed to find any association [13,14]. These contradictory results may due to studies with poor statistical power, A quantitative synthesis with more effective sample size which accumulate data from different studies is needed to provide better evidence on the relationship. In this study, we performed a meta-analysis to determine the association of IL-18 gene polymorphisms (rs1946518 and rs187238 polymorphisms) with RA and SLE risk in Asian populations.

## Methods

### Identification of eligible studies and data extraction

We conducted a computer-based searches of PubMed, EMBASE, Web of Science, China National Knowledge Infrastructure (CNKI),China Biological Medicine Database (CBMD) and Wanfang (Chinese) to identify all studies examining the association of IL-18 promoter rs1946518 and rs187238 polymorphisms with RA and SLE susceptibility with the last report up to April 2012.The following keywords and subject terms were used for searching:“systemic lupus erythematosus”, “SLE”, “rheumatoid arthritis”, “RA”,“IL-18”, “Interlukin-18”and “polymorphism”. References of retrieved articles were also scanned. The following criteria were used to identify the relevant published studies: (i) the diagnosis of RA and SLE was established using the ACR classification criteria for RA and SLE respectively [15-17]; (ii) the study followed a case–control design and the participants were Asian populations with all controls were healthy people; (iii) enough information had to be provided to calculate the odds ratio(OR); (iv) the manuscript was published as a full original paper, not any review or abstract; We excluded the following: (1) studies that contained overlapping data; (2) studies in which the number of wild genotypes could not be ascertained; (3) studies in which family members had been studied because the analysis is based on linkage considerations. For each study we extracted The following information: first author, year of publication, study population (country), the number of patients and controls for the study and genotyping information and frequencies of alleles.

### Statistical analysis

The Hard-Weinberg Equilibrium (HWE) was measured by chi-squared test for the control groups of each study (significance set at *P*<0.05). If the control groups were not in HWE, sensitivity analysis or subgroup analysis was performed to test the robustness of the findings. The odds ratio (OR) corresponding to the 95% confidence interval (95% CI) was used to assess the strength of association between the rs1946518 and rs187238 polymorphisms in the IL-18 gene and the risk of RA and SLE respectively. We performed meta-analyses using (1) allele contrast, (2) dominant, and (3) recessive models. The heterogeneity among studies was assessed using the Q- [18]and I^2^-statistics [19]. Heterogeneity was considered significant for *P*<0.10.If heterogeneity existed, the pooled OR was estimated by the random-effects model and the Dersimonian and Laird method[20], Alternately, a fixed-effect model and the Mantel-Haenszel method were applied [21]. Random effects are more appropriate when heterogeneity is present. The significance of the pooled OR was determined by the Z test. Modified Egger's linear regression test are used to detect publication bias [22]. In addition, population, sample size, genotyping method and status of Hardy-Weinberg Equilibrium were used as characteristics for assessment of heterogeneity, and Meta-regression with restricted maximum likelihood estimation was conducted to explore the potentially important sources of between-study heterogeneity according to above characteristics. Furthermore, subsidiary analyses included subgroup analyses. All analyses were performed by using the software Stata version 10 (StataCorp LP, College Station, TX, USA), All reported probabilities (*P* values) were two-sided.

## Results

### Studies included in the meta-analysis

**Figure 1:**
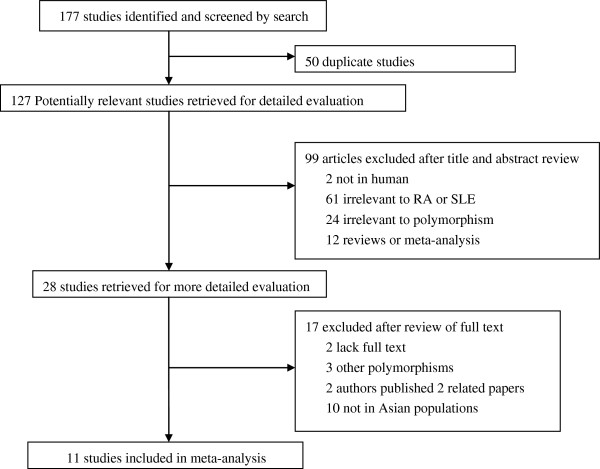
**Flow diagram summarizing the search strategy for meta-analysis of IL-18 gene and RA and SLE**.

A total of 177 articles were identified based on the above searching criteria. 50 duplicate articles were excluded after comparing the title and author name. by reading title and abstracts 99 articles were further excluded (2 were not conducted in humans; 61 were irrelevant to RA or SLE;24 were irrelevant to polymorphism; 12 were review or meta-analysis). After review of full texts of the remaining 28 articles, 17 articles were excluded for failing to meet the inclusion criteria (2 were lack of full text;3 were about other polymorphisms; 2 authors published 2 related reports; 10 were not conducted in Asian populations). Thus, 11 studies were selected in our meta-analysis (Figure 1). Overall, we identified 11 articles with 2805 patients and 2685 controls to evaluate the association of IL-18 rs1946518 and rs187238 polymorphisms with RA and SLE in Asian populations. The characteristics of those studies in the meta-analysis were listed in Table 1.There were 7 studies from China, 2 studies from Singapore, 1 study from Japan and 1 study from Thailand. For the RA studies, one studies were stratified by three independent subpopulations [23]. In most of the studies the polymorphisms in controls were found to occur in frequencies consistent with HWE. However, Strong deviation from HWE was observed for two studies [23,24] (one for the rs1946518 polymorphism in RA and one for the rs187238 polymorphism in SLE).

**Table 1:** Characteristics of studies included in a meta-analysis of the IL-18 polymorphisms and rheumatoid arthritis and systemic lupus erythematosus

**Disease**	**Author(year)**	**Country**	**Population**	**Sample size**	**Polymorphisms evaluated**	**Genotyping**	**HWE in controls**
				**(cases/controls)**			***P*value**
RA	Sivalingam SP.2003 [23]	Singapore	Chinese	309(74/235)	rs1946518	PCR-SSP	<0.001
RA	Sivalingam SP.2003 [23]	Singapore	Chinese	309(74/235)	rs187238	PCR-SSP	0.442
RA	Sivalingam SP.2003 [23]	Singapore	Indian	45(23/22)	rs1946518	PCR-SSP	0.079
RA	Sivalingam SP.2003 [23]	Singapore	Indian	45(23/22)	rs187238	PCR-SSP	0.548
RA	Sivalingam SP.2003 [23]	Singapore	Malay	25(9/16)	rs1946518	PCR-SSP	0.605
RA	Sivalingam SP.2003 [23]	Singapore	Malay	25(9/16)	rs187238	PCR-SSP	0.904
RA	Huang XZ.2007 [11]	China	Chinese	287(119/168)	rs1946518	PCR-SSP	0.783
RA	Huang XZ.2007 [11]	China	Chinese	288(120/168)	rs187238	PCR-SSP	0.728
RA	Sugiura T.2011 [25]	Japan	Japanese	2432(1462/970)	rs1946518	TaqMan	0.052
RA	Ying B.2011 [14]	China	Chinese	360(164/196)	rs1946518	PCR–RFLP	0.543
RA	Shi P.2011 [26]	China	Chinese	207(107/100)	rs1946518	PCR-SSP	0.115
RA	Shi P.2011 [26]	China	Chinese	207(107/100)	rs187238	PCR-SSP	0.677
SLE	Xu Q.2007 [12]	Singapore	Chinese	231(113/118)	rs1946518	PCR–RFLP	0.050
SLE	Xu Q.2007 [12]	Singapore	Chinese	231(113/118)	rs187238	PCR-SSP	0.334
SLE	Lin YJ.2007 [13]	China(Taiwan)	Chinese	301(160/141)	rs1946518	Probe hybridization	0.088
SLE	Lin YJ.2007 [13]	China(Taiwan)	Chinese	307(161/146)	rs187238	Probe hybridization	0.290
SLE	Lan Y.2008 [27]	China	Chinese	275(115/160)	rs1946518	PCR–RFLP	0.606
SLE	Lan Y.2008 [27]	China	Chinese	275(115/160)	rs187238	PCR–RFLP	0.727
SLE	Chen DY.2009 [28]	China(Taiwan)	Chinese	339(165/174)	rs1946518	PCR–RFLP	0.130
SLE	Hirankarn N.2009 [29]	Thailand	Thai	258(116/142)	rs1946518	PCR-SSP	0.061
SLE	Hirankarn N.2009 [29]	Thailand	Thai	258(116/142)	rs187238	PCR-SSP	0.868
SLE	Ye ZZ.2009 [24]	China	Chinese	289(165/124)	rs1946518	PCR-SSP	0.549
SLE	Ye ZZ.2009 [24]	China	Chinese	289(165/124)	rs187238	PCR-SSP	0.030

### Meta-analysis of the IL-18 gene rs1946518 (−607A/C) polymorphism and RA risk

The meta-analysis of the association between the rs1946518 polymorphism and RA risk in Asians (listed in Table 2) was investigated under the additive model (allele A versus allele C), the dominant model (AA+AC vs. CC) and the recessive model (AA vs. AC+CC) . In all genetic models substantial heterogeneity among the studies was found, and random effects model was selected. Meta-analysis showed no statistic association between RA and the IL-18 rs1946518 polymorphism in all genetic models (additive model: OR=0.752,95%CI=0.562-1.006; dominant model: OR=0.730, 95%CI =0.479-1.113; recessive model: OR=0.537, 95%CI=0.271-1.064). Furthermore, we conducted subgroup analysis including population, genotyping method, sample size and HWE status, to analyze characteristic-homogeneous groups in additive genetic model, the results were shown in Table 3. In Chinese population meta-analysis showed an association between RA and IL-18 rs1946518 A allele (OR=0.688,95%CI =0.532-0.889), as well as in studies with PCR-SSP method (OR=0.636, 95%CI =00.519-0.781) and small sample size (OR=0.559, 95%CI =0.438-0.715). When stratifying by HWE, results did not change when restricted to the studies consistent with HWE.

**Table 2:** Meta-analysis of the IL-18 gene polymorphisms and RA risk

**Genetic model**	**Comparisons**	**Test of association**			**Test of heterogeneity**			**Publication bias**
		**OR**	**95 % CI**	***P*value**	**Model**	**I**^**2**^	***P*value**	***P*value**
rs1946518								
Additive	7	0.752	0.562-1.006	0.055	R	77.80%	<0.001	0.065
Dominant	7	0.730	0.479-1.113	0.144	R	74.40%	0.001	0.067
Recessive	7	0.537	0.271-1.064	0.075	R	76.20%	<0.001	0.290
rs187238								
Additive	5	1.316	0.828-2.092	0.246	R	48.90%	0.098	0.845
Dominant	4	1.196	0.466-3.073	0.710	F	45.80%	0.136	0.445
Recessive	5	1.347	0.963-1.885	0.082	F	22.40%	0.272	0.657

R: random model F: fixed model.

**Table 3:** Studies of the rs1946518 and rs187238 polymorphisms in IL-18 gene and risk of RA and SLE under additive model grouped by study characteristics

**Study characteristics**	**Effect estimate of RA**				**Effect estimate of SLE**			
	**Comparisons**	**I**^**2**^	**OR (95% CI)**	***P*value**^**a**^	**Comparisons**	**I**^**2**^	**OR (95% CI)**	***P*value**^**a**^
**rs1946518**	7	77.80%	0.752(0.562-1.006)	0.055	6	88.10%	0.684(0.455-1.028)	0.068
Population								
Chinese	4	52.90%	0.688(0.532-0.889)	0.004	5	87.00%	0.606(0.396-0.930)	0.022
Other	3	40.10%	1.074(0.958-1.205)	0.702	1	NA	1.250(0.883-1.771)	0.208
Genotyping method								
PCR-SSP	5	36.50%	0.636(0.519-0.781)	<0.001	2	96.70%	0.596(0.139-2.560)	0.487
PCR–RFLP	1	NA	0.817(0.604-1.105)	0.189	3	74.60%	0.670(0.454-0.989)	0.044
other	1	NA	1.088(0.969-1.222)	0.155	1	NA	0.945(0.686-1.303)	0.731
Sample size								
≥300	3	50.60%	0.959(0.781-1.178)	0.693	3	76.80%	0.773(0.519-1.152)	0.207
<300	4	0.00%	0.559(0.438-0.715)	<0.001	3	93.50%	0.605(0.267-1.369)	0.228
HWE status								
Meeting HWE	6	81.30%	0.727(0.513-1.031)	0.073	6	88.10%	0.684(0.455-1.028)	0.068
Deviating HWE	1	NA	0.857(0.590-1.244)	0.416	0	NA	NA	NA
**rs187238**	5	48.90%	1.296(0.959-1.751)	0.246	5	33.70%	1.166(0.948-1.435)	0.145
Population								
Chinese	3	70.30%	1.262(0.703-2.264)	0.436	4	45.60%	1.209(0.961-1.520)	0.105
Other	2	0.00%	1.628(0.631-4.199)	0.314	1	NA	0.996(0.614-1.616)	0.986
Genotyping method								
PCR-SSP	5	48.90%	1.296(0.959-1.751)	0.246	3	0.00%	1.300(0.991-1.705)	0.416
Other	0	NA	NA	NA	2	65.10%	0.987(0.570-1.710)	0.963
Sample size								
≥150	3	70.30%	1.262(0.703-2.264)	0.436	2	0.00%	1.351(0.990-1.845)	0.058
<150	2	0.00%	1.628(0.631-4.199)	0.314	3	54.70%	1.040(0.686-1.577)	0.854
HWE status								
Meeting HWE	5	48.90%	1.296(0.959-1.751)	0.246	4	34.20%	1.095(0.870-1.377)	0.439
Deviating HWE	0	NA	NA	NA	1	NA	1.537(0.942-2.508)	0.085

a: *p* value of Z test NA: not available.

### Meta-analysis of the IL-18 gene rs187238 ( −137G/C) polymorphism and RA risk

The summary of meta-analysis for the IL-18 gene promoter rs187238 polymorphism with RA risk in Asians is shown in Table 2. The overall OR under all genetic models were not significant (additive model: OR =1.316, 95%CI =0.828-2.092; dominant model: OR=0.589, 95%CI =0.089-3.903; recessive model: OR=1.116, 95%CI=0.808-1.541). In addition, stratification by study characteristics like population, genotyping method, sample size and HWE status, no significant association was found under the additive genetic model either.

### Meta-analysis of the IL-18 gene rs1946518 (−607A/C) polymorphism and SLE risk

The results of meta-analysis for the IL-18 gene rs1946518 polymorphism and SLE risk in Asians were shown in Table 4. There were no significant association between SLE and the IL-18 -607A/C polymorphism in all genetic models (additive model: OR=0.684, 95%CI=0.455-1.028; dominant model: OR=0.645,95%CI=0.368-1.130; recessive model: OR=0.672, 95%CI =0.447-1.010). When stratification by population and genotyping method in additive genetic model, meta-analysis showed IL-18 rs1946518 A allele might have a protective effect on SLE in Chinese population (OR=0.606, 95%CI =0.396-0.930), and significant association was also found in studies with PCR-RFLP method (OR=0.670, 95%CI =0.454-0.989), but results did not change when stratification by HWE status and sample size.

**Table 4:** Meta-analysis of the IL-18 gene polymorphisms and SLE risk

**Genetic model**	**Comparisons**	**Test of association**			**Test of heterogeneity**			**Publication bias**
		**OR**	**95 % CI**	***P*value**	**Model**	**I**^**2**^	***P*value**	***P*value**
rs1946518								
Additive	6	0.684	0.455-1.028	0.068	R	88.10%	<0.001	0.118
Dominant	6	0.645	0.368-1.130	0.125	R	86.00%	<0.001	0.236
Recessive	6	0.672	0.447-1.010	0.056	R	61.20%	0.024	0.275
rs187238								
Additive	5	1.166	0.948-1.435	0.145	F	33.70%	0.197	0.412
Dominant	5	1.185	0.911-1.543	0.206	F	0.00%	0.961	0.401
Recessive	5	1.688	0.553-5.153	0.358	R	76.40%	0.002	0.561

### Meta-analysis of the IL-18 gene rs187238 ( −137G/C) polymorphism and SLE risk

The fixed model was used to analyze IL-18 gene rs187238 polymorphism with SLE risk under the additive and dominate model, and the random model was performed under the recessive model. No significant association was found under all genetic models (additive model: OR =1.166, 95%CI =0.948-1.435; dominant model: OR =1.185, 95%CI =0.911-1.543; recessive model: OR =1.688, 95%CI =0.553-5.153), and the subgroup analysis also failed to find any significant results.

### Meta-regression analysis

Meta-regression was performed with population (Chinese and other), genotyping method (RFLP and other methods), sample size and HWE status to assess the heterogeneity among the studies. For RA and SLE, when analyzed the rs1946518 polymorphism, strong evidence for heterogeneity among studies was demonstrated under all genetic models. For RA, the regression analysis showed that under the additive model sample size (b coefficient=0.5325109, *P* =0.03, I^2^-residual = 27.30%, adj-R^2^ =80.06%) was a significant source of heterogeneity among studies and under the recessive model population (b coefficient=0.7354299, *P*=0.024, I^2^-residual=0.00%, adj-R^2^ =100.00%) was the significant source of heterogeneity among studies. The other variables were not potentially significant sources of heterogeneity. For SLE, meta-regression failed to identify the sources of heterogeneity.

### Publication bias

We used modified Egger's linear regression test to access publication bias of the rs1946518 and rs187238 polymorphisms in the IL-18 gene and RA and SLE risk respectively under all genetic models (shown in Table 2 and Table 4). The results showed that no statistically significant small study or publication bias was observed (all *P* values for bias >0.05).

## Discussion

A meta-analysis of the relationship of rs1946518 polymorphism with RA and SLE, no significant association with RA or SLE risk can be found under all genetic models in Asian populations, which was similar to the previous meta-analysis reported by Pan et al. [30]. For RA, in the subgroup analysis, results from studies with Chinese population or PCR-SSP method or small sample size were inconsistent. For Chinese population rs1946518 A allele was associated with decreased risk for RA (OR=0.688,95%CI=0.532-0.889), as well as studies with PCR-SSP method (OR=0.636,95%CI=0.519-0.781) and small sample size (OR=0.559,95%CI=0.438-0.715). In addition, subgroup analysis found different characteristics among studies like population, genotyping method, sample size, could explain part of the heterogeneity, but meta-regression analysis can only detect sample size as a significant source of heterogeneity under the additive model. For SLE, meta-analysis showed −607 A allele may have a protective effect on SLE in Chinese population (OR=0.606,95%CI =0.396-0.930) and studies with PCR-RFLP method (OR=0.670,95%CI =0.454-0.989), and neither subgroup analysis nor meta-regression analysis found the sources of heterogeneity under the additive model. Therefore our meta-analysis have not find any association between rs1946518 polymorphism and RA or SLE, but we detected significant association in Chinese population for both diseases, which may be due to different allele frequency at this SNP between Chinese population and other populations, the major populations we investigated were Chinese, Japanese and Thai populations, we compared A allele frequency in Chinese population with Japanese population (HapMap Genome browser release #28) and Thai population (For Thai population we used Hirankarn et al.[29] control data instead ,because we could not get the data from the International HapMap Project). The A allele frequency in Chinese population was 0.585 compared with 0.580 in Japanese population and 0.440 in Thai population. This indicates that the allele frequency does not differ between Chinese population and Japanese population, but differ between Chinese population and Thai population, so it can only explain part of the difference, some other reasons, like genotyping method ,sample size and environmental exposures, may also play important roles. Above analysis suggest that rs1946518 polymorphism existed regional difference,so we should be caution when pooled data in different populations.

However, we failed to find any association between rs187238 polymorphism and RA or SLE under all genetic models in Asian populations, and even in subgroup analysis the results remained unchanged. For both diseases, the rs187238 polymorphism did not show significant heterogeneity among all studies under most genetic models. Considering the limited number of studies included in this study, whether rs187238 polymorphism plays roles in RA or SLE is still unknown, so additional large scale studies about RA or SLE are needed to clarify these relationships.

Rs1946518 and rs187238 polymorphisms in the IL-18 promoter region are associated with transcription activity. A change from nucleotide C to nucleotide A at position −607 disrupts a potential cAMP-responsive element binding protein (CREB) site resulting in higher levels of the IL-18 production [31,32]. For position −137, a change from nucleotide G to nucleotide C affects the H4TF-1 nuclear factor binding site, which may confer a higher IL-18 protein expression [32]. Furthermore, a recent study reported by Dziedziejko et al. [33] also found rs1946518 (−607A/C) AA homozygotes showed significantly lower IL-18 release and mRNA expression after stimulation(stimulants: PHA, LPS, and anti-CD3/CD28 antibodies),and as well as rs187238 (−137G/C) C allele after PHA treatment. These observations support our study, in which IL-18 rs1946518 C allele was associated with high IL-18 production, and high levels of IL-18 were associated with RA and SLE [8,9], so IL-18 rs1946518 A allele might have a protective effect on RA and SLE.

Several limitations of this research should be considered. First, our literature searching was depended on English and Chinese, language bias might be existed. Second, our subjects were all Asians, so it was not suitable to other populations. Third, publication bias may still be present, distorting the meta-analysis. Fourth, we couldn’t calculate the linkage disequilibrium (LD) between SNPs, so we couldn’t exclude the potential likelihood that the significant association in Chinese population was only due to other real significant polymorphisms which were linkage with rs187238. Finally, the studies included were small, our results should be interpreted with caution.

## Conclusions

In conclusion, pooled results demonstrated no significant association between IL-18 polymorphisms and RA or SLE, but subgroup analysis detected a significant association between IL-18 rs1946518 (−607 A/C) polymorphism and both diseases within Chinese population.

## Competing interests

The authors declare that they have no competing interests.

## Authors’ contributions

SC was in charge of collection of data and manuscript preparation. FJ, JR and JL were responsible for checking the data and performing the statistical analysis. WM participated in study design and critically revised the manuscript. All authors were responsible for drafting the manuscript, read and approved the final version.

## Pre-publication history

The pre-publication history for this paper can be accessed here:

http://www.biomedcentral.com/1471-2350/13/107/prepub
